# Characterizing both bacteria and fungi improves understanding of the *Arabidopsis* root microbiome

**DOI:** 10.1038/s41598-018-37208-z

**Published:** 2019-01-10

**Authors:** Joy Bergelson, Jana Mittelstrass, Matthew W. Horton

**Affiliations:** 1 Department of Ecology and Evolution, University of Chicago, Chicago, IL USA; 2 Department of Plant and Microbial Biology, University of Zürich, Zürich, Switzerland

## Abstract

Roots provide plants mineral nutrients and stability in soil; while doing so, they come into contact with diverse soil microbes that affect plant health and productivity. Despite their ecological and agricultural relevance, the factors that shape the root microbiome remain poorly understood. We grew a worldwide panel of replicated *Arabidopsis thaliana* accessions outdoors and over winter to characterize their root-microbial communities. Although studies of the root microbiome tend to focus on bacteria, we found evidence that fungi have a strong influence on the structure of the root microbiome. Moreover, host effects appear to have a stronger influence on plant-fungal communities than plant-bacterial communities. Mapping the host genes that affect microbiome traits identified *a priori* candidate genes with roles in plant immunity; the root microbiome also appears to be strongly affected by genes that impact root and root hair development. Our results suggest that future analyses of the root microbiome should focus on multiple kingdoms, and that the root microbiome is shaped not only by genes involved in defense, but also by genes involved in plant form and physiology.

## Introduction

Bacteria in the plant leaf^1–4^ and root microbiome^5–10^ are influenced by a combination of ecological and environmental factors, and genetic differences among hosts. Laboratory studies have identified plant genes that influence bacteria in the microbiome^1,6^. However, it remains unclear if genes that are tested in laboratory settings are also influential in the wild, or if gene-by-environment interactions have an overriding effect on the microbiome.

A recent study reported that both eukaryotes and bacteria affect the structure of the leaf microbiome^4^. This raises several questions, including: how important are eukaryotes in the root microbiome? Would characterizing eukaryotes help in identifying the environmental and host factors that shape root microbiota? Do the same environmental factors and plant genes shape prokaryotes and eukaryotes?

To address these questions, we sequenced the bacteria and fungi that colonize the root rhizoplane and endosphere of 196 replicated (*n* = 4) accessions of *A*. *thaliana* (Suppl. Table S1). We found that both fungi and bacteria are key members of the root microbiome. In particular, network analyses and principal components analyses (PCA) reveal that, like bacteria, fungi shape the structure of (and variation within) the root microbiome. Furthermore, we found that genetic differences among host plants shape root-microbial communities. The plant genes that are associated with variation in microbiome phenotypes include genes involved in immunity, cell-wall integrity, root, and root-hair development.

## Results

### Comparing the root and leaf microbiome of *A. thaliana*

To characterize root rhizoplane and endosphere bacteria, we amplified and sequenced the hypervariable regions V5, V6, and V7 of 16S ribosomal RNA (rRNA). In addition, we used the fungal primers ITS1F and ITS2 to amplify and sequence the first internal transcribed spacer located within eukaryotic DNA (ITS1). This resulted in ~2524 +/− 1594 (mean +/− s.d.) bacterial reads and 562 +/− 726 fungal reads per sample (Suppl. Fig. S1); sequences sharing more than 97% sequence similarity were clustered into phylotypes.

**Figure 1:**
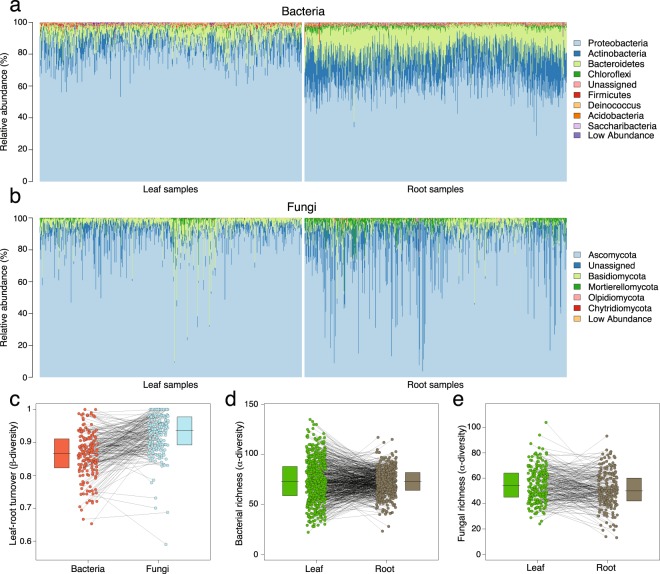
The leaf and root microbiota of worldwide *Arabidopsis* accessions. (**a**) The relative abundances of major bacterial phyla are shown for each leaf and root sample; samples are plotted in columns along the x-axis. (**b**) The relative abundances of fungal phyla are shown for leaf and root samples. (**c**) β diversity in the leaf and root bacterial and fungal microbiome, independent of taxonomic assignments; the lines between points connect samples collected from the same host-plants. The leaf microbial communities of these plants were described earlier. (**d**) Bacterial and (**e**) fungal richness (α diversity) in the leaves and roots. As is the case in (**c**), the lines in (**d**,**e**) connect samples collected from the same host plants.

The soil microbiome forms the starting inocula for roots, and *A*. *thaliana* forms a small (basal) rosette whose leaves regularly come into contact with soil. Therefore, we first asked whether the root and leaf microbiome of *A*. *thaliana* contain similar taxa. Having characterized the bacterial and fungal communities in the leaves of the same plants^2^, we used Poisson generalized linear models to identify differentially enriched (or depleted) taxa in the root microbiome, relative to the leaf. Despite their close physical proximity, we found that the leaves and roots of *A*. *thaliana* differ in the composition of their microbial communities. As an example, roots contain a lower proportion of Proteobacteria than leaves and a correspondingly higher proportion of the phyla Actinobacteria, Bacteroidetes, and Chloroflexi (Fig. 1a). In the case of fungi, Ascomycota and Basidiomycota are more common in the leaf than root microbiome (Fig. 1b), whereas members of the Mortierellomycota are moderately enriched in the root microbiome. Differences among leaves and roots are more pronounced at increasingly specific taxonomic levels; for example, bacterial genera enriched in the root microbiome include the *Massilia*, *Flavobacterium*, and *Actinoplanes*, whereas *Pseudomonas*, *Janthinobacterium*, and *Sphingomonas* are more common in the leaf. In the case of fungi, *Tetracladium*, *Mortierella*, and *Paraphoma* were preferentially associated with roots, whereas *Alternaria*, *Articulospora*, *Cladosporium*, and *Plectosphaerella* were more common in the leaf. Overall, fungi tend to be more difficult to classify at the genus level than bacteria^11^, while microbes from both kingdoms were more difficult to classify in the root than in the leaf microbiome (Table 1).

**Table 1:** The top 10 differentially enriched genera from each kingdom and their preferred habitats.

Kingdom	*Habitat*	*Genus-name*	*Leaf*	*Root*	*Enrichment in root*	P-value
fungi	Root	*Unassigned*	0.370	0.418	1.13	8.98E-197
fungi	Root	*Tetracladium*	0.254	0.280	1.10	1.89E-87
fungi	Leaf	*Alternaria*	0.079	0.049	0.62	0
fungi	Leaf	*Articulospora*	0.064	0.040	0.62	0
fungi	Leaf	*Cladosporium*	0.032	0.026	0.81	1.18E-47
fungi	Leaf	*Plectosphaerella*	0.027	0.021	0.77	1.37E-58
fungi	Root	*Mortierella*	0.007	0.025	3.80	0
fungi	Root	*Paraphoma*	0.015	0.025	1.65	6.81E-167
fungi	Leaf	*Phaeosphaeria*	0.017	0.013	0.74	2.72E-48
fungi	Leaf	*Vishniacozyma*	0.015	0.008	0.52	1.03E-153
bacteria	Root	*Unassigned*	0.174	0.377	2.17	0
bacteria	Root	*Massilia*	0.094	0.257	2.74	0
bacteria	Leaf	*Pseudomonas*	0.219	0.052	0.24	0
bacteria	Leaf	*Janthinobacterium*	0.174	0.000	0.00	0
bacteria	Root	*Flavobacterium*	0.058	0.145	2.48	0
bacteria	Leaf	*Sphingomonas*	0.105	0.017	0.16	0
bacteria	Leaf	*Rhizobium*	0.057	0.012	0.20	0
bacteria	Root	*Actinoplanes*	0.008	0.032	3.94	0
bacteria	Root	*Devosia*	0.008	0.010	1.30	7.76E-113
bacteria	Leaf	*Methylobacterium*	0.009	0.001	0.16	0

The estimates (coefficients) and *P* - values are from a generalized linear (Poisson) model, fit to determine the effect of the factor organ while taking into account differences in sequencing effort among samples. Note: the ‘Unassigned’ categories include diverse taxa, which themselves may be differentially enriched (or depleted) in the root relative to the leaf microbiome.

To further investigate differences in the leaf and root microbial communities, independently of taxonomic assignments, we estimated Whittaker’s β diversity^12^ across the paired leaf and root microbiome of each host plant. On average, leaf and root bacterial communities tend to be more similar than leaf and root fungal communities (Fig. 1c). Comparing samples *within* each organ further revealed that β diversity tends to be higher in the root (Whittaker’s $\bar{{\rm{\beta }}}$ bacteria: 0.87; fungi: 0.91) than leaf (Whittaker’s $\bar{{\rm{\beta }}}$ bacteria: 0.67; fungi: 0.89) microbiome (Suppl. Fig. S2).

Analyses of α diversity revealed that richness is remarkably similar across plant organs. For example, we found no evidence that the leaf and root microbiome differ in fungal richness (Poisson generalized linear mixed-model, GLMM; *P* = 0.62). Although richness in the bacterial community was significantly higher in the leaf than root-microbiome (*P* < 2.2 × 10^−16^), the effect is small (Fig. 1d) and consistent in magnitude with prior insignificant results^13^. What is clear is that richness is higher in the bacterial than fungal community in both the leaf and root microbiome (Fig. 1d,e).

### The structure of the microbiome

The strongest correlations among the best-sequenced (the ‘top 100’) taxa in the plant microbiome tend to occur between members of the same kingdom (Suppl. Fig. S3a,b). In the bacterial community, for example, phylotypes of Comamonadaceae and Massilia showed the highest positive mean correlations with other bacteria in the leaf and root microbiome, respectively. Comamonadaceae has been reported to be a keystone member of the leaf microbiome of *A*. *thaliana* plants grown in Europe^4^; our results indicate it has a similar role in North America. In the case of fungi, two distinct Articulospora phylotypes showed the highest positive mean correlations with other fungi in the leaf and root microbiome.

We found strong and significant cross-kingdom correlations, which raises the possibility that bacteria and fungi interact or that they are shaped by similar processes (environmental and/or host factors). The highest positive mean cross-kingdom correlation was observed in the root microbiome, occurring between fungi and a bacterial phylotype assigned to Flavobacterium (mean *r* = 0.09; range: −0.35 < *x* < 0.93). On average, both intra- and inter-kingdom correlations exhibited a slight positive skew in the leaf and root microbiome (Suppl. Fig. S3c,d); strong negative correlations, which would be consistent with antagonistic effects, do not appear to be common. The highest negative mean intra-kingdom correlation (mean *r* = −0.03) was observed for a Pseudomonas phylotype and its correlations with other bacteria. The highest negative mean cross-kingdom correlation (mean *r* = −0.027) was observed between fungi and a Microbacteriaceae phylotype.

**Figure 2:**
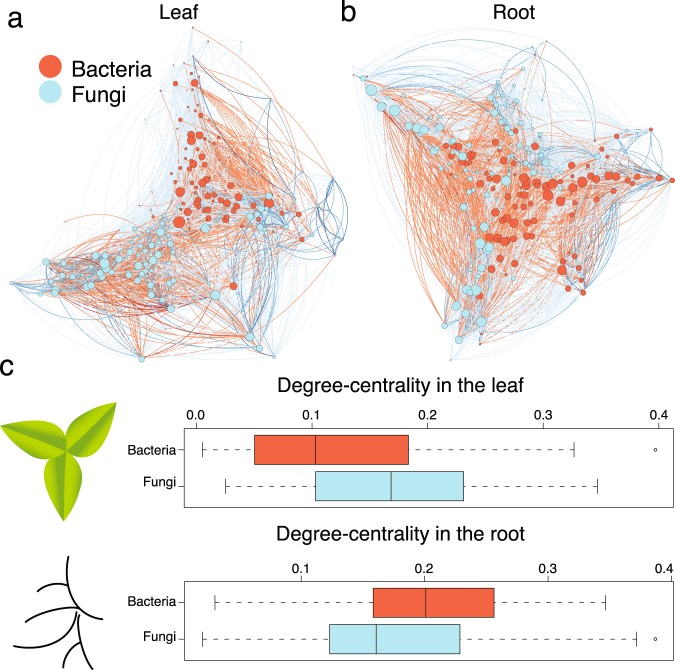
Network analyses of the root and leaf microbiome. Network analyses of Pearson correlation coefficients reveal that both bacteria and fungi are key taxa in the (**a**) leaf and (**b**) root microbiome. The size of each node represents its degree; its color represents its kingdom (blue for fungi, red for bacteria). Edges (lines) between nodes are colored blue for positive correlations between taxa; negative correlations are colored red. The networks are plotted using a Davidson-Harel layout. (**c**) Fungi have more connections (measured by degree centrality) than bacteria in the leaf microbiome, but a similar number of connections in the root microbiome.

Next, we performed network analyses of these correlation coefficients for the leaf and (separately) root microbial community, which revealed that both bacteria and fungi contribute to the structure of these communities. As an example, using measures of centrality to determine the relative importance of bacteria and fungi (represented by their nodes) in each network indicates that fungi are more central to the structure of the leaf microbiome than bacteria (Fig. 2a), as fungi tend to have a higher number (that is, degree) of network connections than bacteria (simple linear model, *P* = 0.00038). In contrast, bacteria and fungi in the root microbiome (Fig. 2b) appear to have roughly the same number of network connections (*P* = 0.09; Fig. 2c). The taxa with the highest degree and betweenness centrality in the leaf and root microbiome are illustrated in (Suppl. Fig. S3e,f).

**Figure 3:**
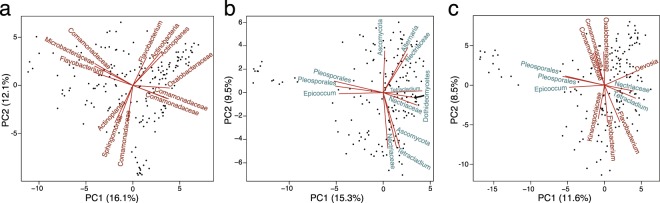
PCA reveals the structure of the root microbiome. (**a**) A plot of principal component 1 (PC1) and PC2 from PCA of the bacterial community. (**b**) A plot of PC1 and PC2 from PCA of the fungal community. (**c**) A plot of PC1 and PC2 from PCA of the combined bacterial and fungal community. In each panel, the labels list the top 3 taxa that separate the samples along each axis (the lines represent their PC loadings).

PCAs of the top 100 root bacteria and, separately, the top 100 fungi suggest that bacteria and fungi may interact or that they are shaped by similar processes. That is, Procrustes analysis of these two separate PCAs (PCs 1–3) revealed that these communities have similar but not identical community structure (*r = *0.4, *P = *0.001; expanding this to 10 PCs results in *r* = 0.497, *P* = 0.001; *n* = 999 permutations). To further investigate these patterns, we combined the top 100 taxa from each kingdom into one microbiome and then repeated PCA. Figure 3 shows the first two axes from separate PCAs of root bacteria, root fungi, and the combined root bacterial and fungal community. Also shown are the top taxa that separate the samples in each analysis. As illustrated in Fig. 3c, five of the top six taxa that separate samples along PC1 from PCA of the combined community are fungi, which suggests that between individual variation in the root microbiome is heavily shaped by fungi.

While PC1 from PCA of the combined community is strongly correlated with PC1 from the fungal community (*r* = 0.94; Suppl. Fig. S4), PC2 is correlated with PC1 of bacteria (*r = *0.93). Indeed, four of the six taxa that separate samples along PC1 from bacteria (Fig. 3a) separate samples along PC2 from the combined community (Fig. 3c). The situation is reversed in the leaf microbiome, where variation along PC1 from bacteria is represented by PC1 from the combined community (*r = *0.94), while PC1 from fungi separates samples along PC2 of the combined community (*r = *0.96; Suppl. Fig. S4). The leaf bacteria that distinguish samples along PC1 from the combined community include members of the *Sphingomonas*, Sphingomonadales, and a *Methylobacterium* (Suppl. Fig. S5), alphaproteobacteria that have been observed in the phyllosphere of several plant species^14^.

### The root microbiome is shaped by host-genetic variation

Genetic differences among hosts influence the composition and diversity of both animal^15,16^ and plant-associated^17,18^ microbial communities. In the case of the rhizosphere, genetic differences among host plants are known to influence the abundance of bacteria^17^, the number of bacterial taxa^7^, and the structure of the bacterial community^5^. However, it is unclear whether fungi in the root microbiome of *A*. *thaliana*, which is non-mycorrhizal, are also shaped by plant genes and, if so, to what extent. It also remains unclear whether host factors independently shape root bacteria and fungi, or if host effects operate at the level of the combined microbiome.

**Figure 4:**
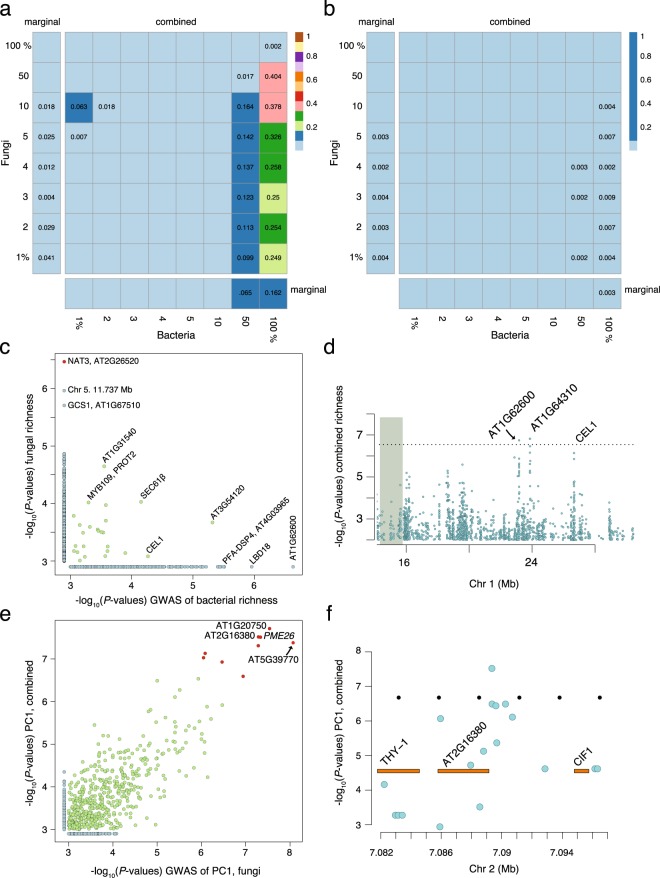
Evidence that hosts shape their microbiome is stronger when taking into account cross-kingdom correlations. (**a**,**b**) The color and number in each square represents the *P*-value from (*n* = 999) permutation tests that investigate whether genetic differences among accessions shape the bacterial and fungal communities independently or as a combined microbiome (see Methods). The labels along the margins indicate how much of the community was considered in each analysis (e.g. top 1% refers to the top 1% best-sequenced taxa) and is sorted in decreasing abundance. The bars along the margins report the results from single-kingdom (marginal) analyses, while the grids in each panel show the results from the combined (bacteria + fungi) community analyses. The *P*-values in each square are shown when *P* > 0.001 (that is, empty squares occur when *P* = 0.001). The plot in (**a**) shows the results from analyzing the first three PCs, while (**b**) shows the results from analyzing the first five PCs. (**c**) The overlap in the results (10-kb windows) from GWAS of bacterial and fungal richness. (**d**) The results from GWAS of richness in the combined bacterial and fungal community along the lower arm of chromosome 1. (**e**) The overlap in the results (10-kb windows) from GWAS of PC1 from PCA of the fungal community (x-axis) and the combined fungal and bacterial community (y-axis). (**f**) The results from GWAS of PC1 from PCA of the combined microbiome include a peak on chromosome 2 that falls between AT2G16380 and CIF1. For (**c**,**e**) significant results are shown in red. All GWAS were performed using a linear mixed model in order to adjust/account for confounding due to population structure.

To examine the role of host plants in shaping variation in the microbiome on the rhizoplane and in the endosphere, we asked whether the microbial communities of inbred *Arabidopsis* accessions cluster together in the results from PCA. Evidence that the bacterial community differs among accessions was restricted to the best-sequenced taxa, which is consistent with the results from our earlier analysis of the leaf microbiome^2^. In contrast, we found clear evidence that the fungal community differs among accessions. Remarkably, combining the bacterial and fungal community together before PCA provided evidence that host plants also shape their combined root-associated microbial communities (Fig. 4a,b) although this was not evident for the leaf microbiome.

Next, we estimated the proportion of variation in the microbiome explained by genetic relatedness among accessions (Methods), the latter which we estimated using ~1.8 million genome-wide single nucleotide polymorphisms (SNPs). We found that host plants shape the root microbiome in a variety of ways, including richness in the bacterial community (SNP-*h*^2^ ~ 0.21), fungal community (SNP-*h*^2^ ~ 0.52), and the combined bacterial and fungal community (SNP-*h*^2^ ~ 0.40). The discovery that SNP heritability is higher for richness in the fungal than bacterial community prompted us to reanalyze richness in the leaf microbiome using these dense SNP data; again, we found that SNP-*h*^2^ of richness is higher for fungi (SNP-*h*^2^ ~ 0.25) than bacteria (SNP-*h*^2^ ~ 0.15). Estimates of broad-sense heritability, however, indicate that the opposite is true (Table 2). The discrepancy may be due to differences in genetic architecture for the bacterial and fungal components of the plant microbiome. In particular, SNPs may underestimate genetic variance for traits influenced by non-additive effects or low-frequency causal SNPs in incomplete linkage disequilibrium with sequenced SNPs^19^; genetic heterogeneity may pose additional problems for SNP-based approaches if bacteria and fungi are differentially affected by functionally redundant members of large gene families, including ATP-binding cassette (ABC) transporters and leucine-rich repeat (LRR) genes implicated in defense^2^. Conversely, estimates of narrow-sense heritability calculated using best-linear unbiased predictors (BLUPs) may outperform estimates of broad-sense heritability when samples (individuals) within an inbred line are not equally colonized. In such a situation, estimates of narrow-sense heritability benefit from investigating a host’s ‘average’ microbiome, which is not unlike the common approach to pool samples before analysis.

**Table 2:** Estimates of broad (*H*^*2*^) and narrow-sense heritability (*h*^*2*^) of richness in the root and leaf microbiome of *A*. *thaliana*.

*Organ*	*Microbiome trait*	*H* ^*2*^	*h* ^*2*^
root	bacterial richness	0.57	0.21
root	fungal richness	0.47	0.52
root	combined bacteria and fungi	0.29	0.40
leaf	bacterial richness	0.76	0.15
leaf	fungal richness	0.51	0.25
leaf	combined bacteria and fungi	0.31	0.19

Both broad and narrow-sense estimates were calculated from a GLMM that corrected for sequencing effort and other technical artifacts. SNPs (minor allele frequency, or MAF ≥ 0.05) from the 1001 Genomes Project^44^ were used to estimate narrow-sense heritability.

### The plant genes that shape the root microbiome

Finally, we turned to identifying the host genes that influence the root microbiome. To do so, we used GWAS to identify the major genetic variants that underlie variation in the microbiome traits species richness and community structure, the latter which we characterized using PCA. In addition, to better understand the biological processes shaping each trait, we identified gene ontology (GO) categories enriched (FDR *q* < 10%) in the results from GWAS^20^.

First, we asked whether the plant genes that shape richness in the bacterial community also affect the fungal community. For example, the top result from GWAS of bacterial richness falls within (Chr 1, 23.179 Mb, *P* = 2.33 × 10^−7^), an as yet undescribed flavin monooxygenase gene (*AT1G6*2*600*). A related flavin monooxygenase gene, *FMO1*, was recently shown to play a crucial role in plant immunity in the leaf by hydroxylating and converting pipecolic acid into the systemic signaling molecule N-hydroxypipecolic acid^21^. The second strongest peak of association falls on chromosome 2 (18.727 Mb, *P* = 1.1 × 10^−6^) immediately 3′ of Lateral Organ Boundary domain-containing protein 18 (*LBD18*); *LBD18* is involved in the initiation and emergence of lateral roots^22^. The top candidate genes from GWAS of bacterial and fungal richness are shown in Fig. 4c. Although the bacterial richness candidates failed to reach genome-wide significance, GWAS of richness in the combined (bacterial + fungal) microbiome identified a few of the same candidates including *AT1G6*2*600* (Fig. 4d).

Overall, the results from GWAS of bacterial and fungal richness showed little overlap. While this suggests that richness in the bacterial and fungal communities are influenced by different plant genes, a few interesting candidates were identified in both analyses. A subunit of *SEC 61β* (Chr 5, 24.318 Mb, *P* < 1 × 10^−4^), a component of the SEC 61 (translocon) protein channel, was among the most promising (Fig. 4c). Molecular analyses of the SEC 61 channel have demonstrated that *SEC 61β* acts as the point of contact with SNARE proteins that are critical to protein transport in root hairs; moreover, disruptions in this pathway result in reduced root hair length^23^. Research in barley has reported that *SEC 61β* also plays a role in leaf-microbial interactions, as silencing *SEC 61β* leads to an increase in plant-resistance by disrupting contact with fungal haustoria^24^.

On average, bacterial richness is higher than fungal richness in the microbiome of *A*. *thaliana* (Fig. 1d,e). To determine if accessions of *A*. *thaliana* differ in the ability to be colonized by bacteria and fungi, and if some accessions are even more amenable to bacterial colonization, we calculated the difference in species richness between the two communities (Methods). We found that the ‘preference’ of plants to host diverse bacterial rather than fungal (or vice versa) communities is shaped by genetic differences among hosts (SNP-*h*^2^ ~ 0.59). The top SNP from GWAS (Chr 2, 6.09 Mb, *P* = 6.86 × 10^−7^) falls alongside an as yet undescribed member (*LCR84*) of a gene family believed to have a role in innate immunity. The top results also include the Nucleobase ascorbate transporter (NAT) 3 locus and a NBS-LRR disease resistance gene (*AT1G31540*) that is also associated with fungal richness (Fig. 4c).

Gene set enrichment analyses helped to identify the underlying biological processes shaping microbiome traits. For example, the top GO categories enriched in the results from GWAS of bacterial preference relate to root development (radial pattern formation, root morphogenesis), pectate activities, vasculature, and aging (Table 3). The biological processes associated with bacterial richness relate to cell-wall modification, sugar processing, and cellulase activities, while processes related to the epidermal cell layer and programmed cell death underlie variation in fungal richness (Suppl. Table S2).

**Table 3:** The biological processes that underlie variation in the root microbiome.

*Biological process*	*enrichment*	*FDR q < 10%*
stomatal complex formation	77.4	0.0004
PSII associated light-harvesting complex II catabolic process	18.1	0.0035
leaf vascular tissue pattern formation	7.3	0.0244
radial pattern formation	14.7	0.0277
root morphogenesis	13.4	0.0277
Golgi transport complex	12.9	0.0277
DNA endoreduplication	7	0.0355
negative regulation of photomorphogenesis	10.3	0.0355
photoinhibition	10.3	0.0355
embryo sac development	3.7	0.0675
pectate lyase activity	5.3	0.0683
anther dehiscence	7.2	0.0737
cellular response to abscisic acid stimulus	6.4	0.0843
nitrogen compound metabolic process	6	0.0843
cotyledon development	4.4	0.0843
cyclin-dependent protein kinase activity	4.4	0.0843
aging	3.6	0.0843

The biological (gene ontology, GO) processes enriched in the 1% tail results from GWAS, after mapping the difference in species richness in the bacterial and fungal communities for each accession. Storey’s procedure^20^ was used to correct for multiple testing.

Like the leaf microbiome^2^, the root microbiome appears to be influenced by plant genes responsible for cell-wall integrity. In three separate GWAS of PC2 from PCA of root bacteria, PC1 for fungi, and PC1 of the combined microbiome, we found the candidate gene *PECTIN METHYLESTERASE 26* (*PME26*) and its neighbor *PME3*. The peak within *PME26* puts it among the top 3 candidates from GWAS of PC1 from fungi and the combined microbiome (Fig. 4e); it is the top result from GWAS of PC2 from PCA of bacteria.

The strongest peak of association from PC1 from PCA of the combined community falls inside a gene (Chromosome 1, 7.2 Mb; *P* = 1.93 × 10^−8^) that encodes a RAD3-like DNA-binding helicase (*AT1G20750*); this gene is surrounded by a pair of F-box genes and other genes involved in the trans-golgi network. A possibly related peak of association was found on chromosome 5 (*P = *4.17 × 10^−8^) and falls within *AT5G39770*, a homolog of the crossover junction nuclease *MUS81*. Although *AT5G39770* was annotated as a pseudogene in the reference genome, it appears to be expressed in natural accessions^25^. It remains unclear how *AT5G39770* might impact the microbiome, but in the mutant background of a different RAD3 domain-containing helicase (namely, *RTEL1*) *MUS81* mutants exhibit delayed and aberrant root development^26^.

Of the three remaining significant peaks of association (Chr. 2, 7.089 Mb, *P* = 3.03 × 10^−8^; Chr 3, 13.802 Mb, *P = *4.88 × 10^−8^; Chr 3, 16.555 Mb, *P = *1.17 × 10^−7^; Suppl. Fig. S6), the strongest is on chromosome 2 and falls between (Fig. 4f) two promising candidate genes: *AT2G16380*, a homolog of *SHORT ROOT HAIR 1* (*SRH1*), and *CASPARIAN STRIP INTEGRITY FACTOR 1* (*CIF1*). The Casparian strip is a hydrophobic layer in the root endodermis that acts to regulate the flow of water and ions between the soil and vascular tissue; formation of the diffusion barrier requires the expression of the peptide hormones CIF1 and CIF2^27,28^.

## Discussion

We characterized the bacteria and fungi found in the root microbiome of genetically diverse *A*. *thaliana* accessions. We found that the root endosphere and rhizoplane are colonized by diverse bacteria and fungi (Fig. 1), and that variation within the leaf and root microbiome is influenced by members of both kingdoms (Figs 2 and 3, Suppl. Figs S3 and S5).

Despite widespread interest in root-bacterial communities, fungi also play a key role in the root microbiome (Fig. 3c), especially as it relates to host genetics (Fig. 4a,b and Table 2). We interpret this as evidence that previous studies of the root microbiome (and perhaps other microbial communities) were limited when focusing only on bacteria, while noting that other microorganisms and viruses are also likely to be of interest.

One of the main challenges in characterizing a microbiome is indeed determining how to do so in a biologically relevant manner. For one thing, taxa within the microbiome interact with one another, which weakens the rationale to treat individual microbes as independent. In addition, RNA operon counts differ among species, which precludes accurate estimates of abundance. Even worse, it is difficult to infer the function of the microbiome due to horizontal gene transfer, because taxa with identical RNA genes can possess very different genomes^29^. The latter two problems are of particular concern when investigating taxonomically or geographically diverse samples, when short-read sequencing technologies and low genetic variation at phylogenetic markers are already expected to obscure relevant differences among strains.

Despite the clear need to identify factors that shape the plant microbiome, our understanding of these communities lags behinds knowledge of other important microbes, such as those that colonize the human gut. Regardless of the technique that we used, we found that the structure and diversity of the root microbiome is shaped by genetic differences among accessions. What’s more, using GWAS, we were able to identify excellent candidate genes associated with diversity and microbial community structure (Fig. 4c–f). As an example, GWAS of richness in the combined bacterial and fungal microbiome identified an undescribed member of the flavin monooxygenase gene family (*AT1G62600*). It was recently demonstrated that a related flavin monoxygenase, *FMO1*, plays a fundamental role in systemic immunity in the leaves of *A*. *thaliana*^21^; the results presented here raise the possibility that *AT1G62600* plays a similar role in shaping root-microbial communities. In addition to identifying candidate genes with putative roles in immunity, we also identified candidate genes involved in cell-wall integrity, root, and root-hair development. These results indicate that the root microbiome is not only shaped by immune-related loci, but also by genes that determine plant form and physiology. Experiments to investigate the most promising candidates are currently underway.

The genetic architecture of the root microbiome is complex. Environmental factors (e.g. UV, rainfall), host-nutrient status, and whether or not a given microbe occurs in a particular habitat all influence efforts to identify the factors that shape root microbiota. In human genetics, non-genetic factors (e.g. age, smoking status, body mass index) are regularly included as covariates to investigate diseases; it is likely that controlling for environmental variation (e.g. soil chemistry) will likewise improve our understanding of the root microbiome.

Because this experiment was conducted as a proof of concept, we controlled for environmental variability (Methods) by growing these accessions in a field site known to host *Arabidopsis*. However, it is tempting to speculate that, due to environmental variation, different loci would be mapped if this experiment were repeated at a different time or in a different place. The same is true of phenotypes believed to be highly heritable, including flowering time^30–32^. Nevertheless, one of the main reasons to investigate the plant microbiome is because of its role in plant health and productivity. In agricultural efforts, the environment can be, and usually is, managed. Our results reveal that GWAS of the plant microbiome, a complex multi-kingdom community, will be a powerful approach in such situations.

## Methods

### Plant material

We sowed four seeds from each of 196 worldwide accessions of *Arabidopsis thaliana* (L.) Heynh in a randomized block design, watered them, and then placed them in a cold (4 °C) dark room for seven days to homogenize germination. These plants were then moved to a glasshouse where they were grown for 19 days before being transferred to a field site known to host a wild population of *A*. *thaliana* (42.0831N, 86.351W). After growing in the field for ~five months (156 days), the leaves and roots of each plant were collected using sterile technique and then flash-frozen in liquid N_2_. The leaf microbial communities from these plants were described earlier^2^.

### Amplicon preparation and sequencing

During this study, root DNA was extracted using MoBio’s (now Qiagen) PowerSoil DNA isolation Kit (MoBio Laboratories, Carlsbad, CA, USA). To increase DNA yield, we repeated the manufacturer’s recommended freeze-thaw process three times before DNA extraction. All other steps, including the PCR/sequencing conditions, denoising steps, chimera removal, and the strategy used to identify species-level phylotypes (so-called operational taxonomic units or OTUs) were performed as described earlier^2^, and are briefly described here. To characterize fungal communities, we amplified ITS1 using the PCR primers ITS1F^33^ and ITS2^34^. To characterize bacterial communities, we amplified the hypervariable regions V5-7 of 16S rRNA, using the primers 799 F^35^ and 1193R^13^. ITS1F and ITS2 exclude host-plant DNA; to avoid sequencing host-plant mtDNA generated with the bacterial primers 799F and 1193R, we ran all PCR products on 2% agarose gels before excising and extracting the phylogenetic target (~505 bp, including phylogenetic adaptors) using Qiagen’s QIAquick gel extraction kits. All amplicon libraries were quantified using a Qubit dsDNA HS Assay Kit (Invitrogen) before being sequenced using 454 FLX Titanium based chemistry (Roche Life Sciences). After sequencing, the data were denoised using AmpliconNoise (version 1.25) through QIIME^36^. Reads longer than 500 bp were discarded and *Perseus* was used to remove chimeras. To identify phylotypes, we used QIIME’s (1.3) implementation of the algorithm cdhit (3.1), while requiring a nucleotide sequence similarity threshold of 97%.

To identify taxa in the root microbiome – and to update the taxonomic assignments of the leaf microbiome – we used SILVA123_QIIME_release for bacteria^37^ and the UNITE database for fungi^38^. The phylotype names used throughout the text refer to the lowest-level assignments obtained through Silva and UNITE. While performing quality control, we discovered sequences that were unassigned at the kingdom level, assigned to Chloroplast at the class level, or assigned to Mitochondria at the family level. These sequences and all singleton taxa (defined here as taxa observed in only one sample) were excluded from downstream analyses; the remaining phylotypes were analyzed as described below.

### Analyses of alpha and beta diversity

Unless otherwise stated (e.g., during data visualization), we explicitly added offset variables in generalized linear models and mixed-models (GLMs, GLMMs) to correct for differences in sequencing effort/coverage among samples. While visualizing data (that is, while creating Figs 1c–e, 2, S2 and S3), we corrected for differences in sequencing effort by resampling the data; the technique is described below.

Richness and microbial abundance data are represented by count data; therefore, we used Poisson GLMs and GLMMs during analysis. In addition, Poisson GLMs were used to identify differentially enriched taxa in the leaf or the root microbiome. During the latter analysis, the factors block and sequencing run were included as fixed effects and, to take into account differences in sequencing effort among samples, the log number of reads in each sample was included as an offset. These models were fit using the native R function *glm*^39^.

To investigate richness (α diversity) in each community, we extended this model to include the number of taxa (phylotypes or OTUs) as the response variable and the host-genotype identifier as a random effect. These Poisson GLMMs were fit using the function *glmer*, which is available in the R-package lme4^40^. To investigate differences in α diversity across accessions, we extracted the best-linear unbiased predictors (BLUPs; the conditional modes of the random effects) from these models using the function *ranef*. To investigate species richness in the combined bacterial and fungal community, we combined data from each kingdom and specified kingdom as a fixed effect, with the sequencing run identifier nested in kingdom. All of the code is available (see Data and materials).

The overall species turnover between and within the leaves and roots was modeled using Whittaker’s estimate of β diversity^12^. To allow differences among samples in α and β diversity to be visualized (that is, when creating Fig. 1c–e), each sample was resampled once to contain 400 reads, using the raw frequency distribution observed in each sample. This relatively low read-count reflects the lower sequencing effort (and higher multiplexing) used to characterize diversity within the fungal community (Suppl. Fig. S1). However, the observed patterns, higher α diversity within the bacterial community and higher β diversity within the fungal community were not affected by the sequencing threshold used (e.g. n = 250, 400, 500, 1000 reads) during resampling. Although lower sequencing thresholds underestimate α diversity and tend to overestimate β diversity (a boundary problem as β diversity approaches one), the relative differences between the bacterial and fungal communities observed during data visualization were consistent with the statistical analyses, during which (as described above) we used offset variables (instead of rarefaction/resampling) to correct for differences in sequencing coverage per sample.

### The structure of the microbiome

We used the function *rcorr* (type = “Pearson”) to calculate and assess the significance of correlations among taxa after adjusting for differences in sequencing effort among samples (see below); the function *rcorr* is available through the R-package Hmisc^41^. We performed network analyses on these correlation coefficients (*P* < 0.01) using the R-package igraph^42^. Influential nodes were identified based on their degree and betweenness, which we calculated using igraph’s functions of the same name.

Although data normalization (standardization) is common during network analyses of gene expression, it does not appear to be widely used during network analyses of microbial communities. We investigated whether correcting for differences in sequencing effort (by resampling or rarefying) affects the construction of microbial community networks. To do so, we compared the centrality metrics of networks constructed using different (minimum) sequencing thresholds. We found that differences in the number of sequences among samples affect inference about node centrality (including node degree); we therefore resampled these data to 400 reads per sample to create the networks depicted in Fig. 2 and the correlation matrix shown in Fig. S3 (using the resampling approach described above). Increasing the minimum read count threshold (e.g. 1000 reads per sample) reduced the number of analyzed samples (due to the lower sequencing effort used to characterize the fungal community), but did not qualitatively affect the results.

In separate analyses, we investigated correlations among bacteria, fungi, and then the composite microbiome (bacteria and fungi) using Poisson GLMMs to model the abundance of the top 100 taxa in each kingdom. As described above, the BLUPs from these GLMMs were extracted with the lme4 function *ranef* and then analyzed using PCA, using the native R function *prcomp* (center and scale = TRUE). The function *protest*, found in the R-package vegan^43^, was used to perform Procrustes analysis.

### The effect of the host

Three independent approaches were used to investigate whether the structure of the microbiome is shaped by genetic differences among hosts. First, we started by sorting the separate bacterial and fungal species matrices in decreasing abundance and sampling these two communities in increasingly inclusive subsets (top-1%, top-2%, …, 100%); these sample subsets were then analyzed with PCA, using different numbers of PCs in both marginal and, separately, combined (bacteria and fungi) analyses. To determine whether replicates of inbred lines cluster together in the multidimensional ordination space formed by PCA, we used vegan’s functions *rda* (scale = TRUE) and *envfit*. During each analysis, we corrected for block effects and technical artifacts due to sequencing.

In separate analyses, we investigated whether genetic similarity (that is, kinship) among accessions explains variability in the microbiome^19^; the phenotypes chosen to represent the microbiome were (1.) species richness and (2.) the coordinates of each accession along the individual PCs from PCA (above). Genetic similarity (identity by descent, or IBD) among accessions was estimated using ~1.8 M SNPs (MAF > 0.05) sequenced during the 1001 Genomes Project^44^. To estimate broad-sense heritability, we calculated the difference between the conditional and marginal variance^45^ for these mixed-models using the function *r*.*squared*.*merMod*, which is maintained in the R-package piecewiseSEM^46^.

To map the major genetic variants associated with variation in the microbiome, we performed genome-wide association studies (GWAS), using linear mixed models to correct for confounding due to population structure^47^. The microbiome traits investigated with GWAS were (1.) species richness and, separately, (2.) the PCs from PCA of the top 100 bacteria, the top 100 fungi, and then the top 100 taxa from each community in a combined analysis. For species richness, we performed GWAS on the BLUPs from the GLMMs described above. For PCs from PCA, we modeled the abundance of each taxon (phylotype) using Poisson GLMMs and then performed PCA on the BLUPs from these models. To estimate a genome-wide significance threshold, we performed permutations while controlling for population structure by linearly transforming the phenotype values using a Cholesky decomposition of the (inverse) phenotypic covariance matrix. We described this approach and our approach for conducting gene set enrichment analyses earlier^2^.

## Supplementary information

Supplementary Info

## Data Availability

The sequencing data have been deposited in the European Nucleotide Archive (ENA) under accession code: *PRJEB27774*. The code to conduct GWAS is available at: https://github.com/bvilhjal/mixmogam. The phenotypes and GWAS results are available at the Dryad Digital Repository (doi:10.5061/dryad.n7n170m). The custom R-scripts are available online at https://github.com/mahort/root_microbiome.
