# Supplementary material for: Characterizing both bacteria and fungi improves understanding of the Arabidopsis root microbiome
Source: Sci Rep. 2019 Jan 10;9:24. doi: 10.1038/s41598-018-37208-z (PMC6328596; doi:10.1038/s41598-018-37208-z)
Supplement: Supplementary file 1 — Supplementary Info [file 41598_2018_37208_MOESM1_ESM.pdf]

Characterizing both bacteria and fungi improves understanding of the *Arabidopsis* root microbiome

Supplementary Material.

Joy Bergelson, Jana Mittelstrass, Matthew W. Horton

This file includes:

Supplementary Figures 1-6:

Fig. S1, Read counts per sample.

Fig. S2, β-diversity in the microbiome

Fig. S3, Key taxa in the microbiome

Fig. S4, Correlations among the PCs from PCA of the microbiome

Fig. S5, The results from PCA of the leaf microbiome

Fig. S6, GWAS of PC1 from PCA of the combined microbiome

Supplementary Tables S1 and S2:

Table S1, The list of accessions used and their collection locations

Table S2, The biological processes that underlie variation in richness in the root microbiome

<u>**Supplementary Figures**</u>

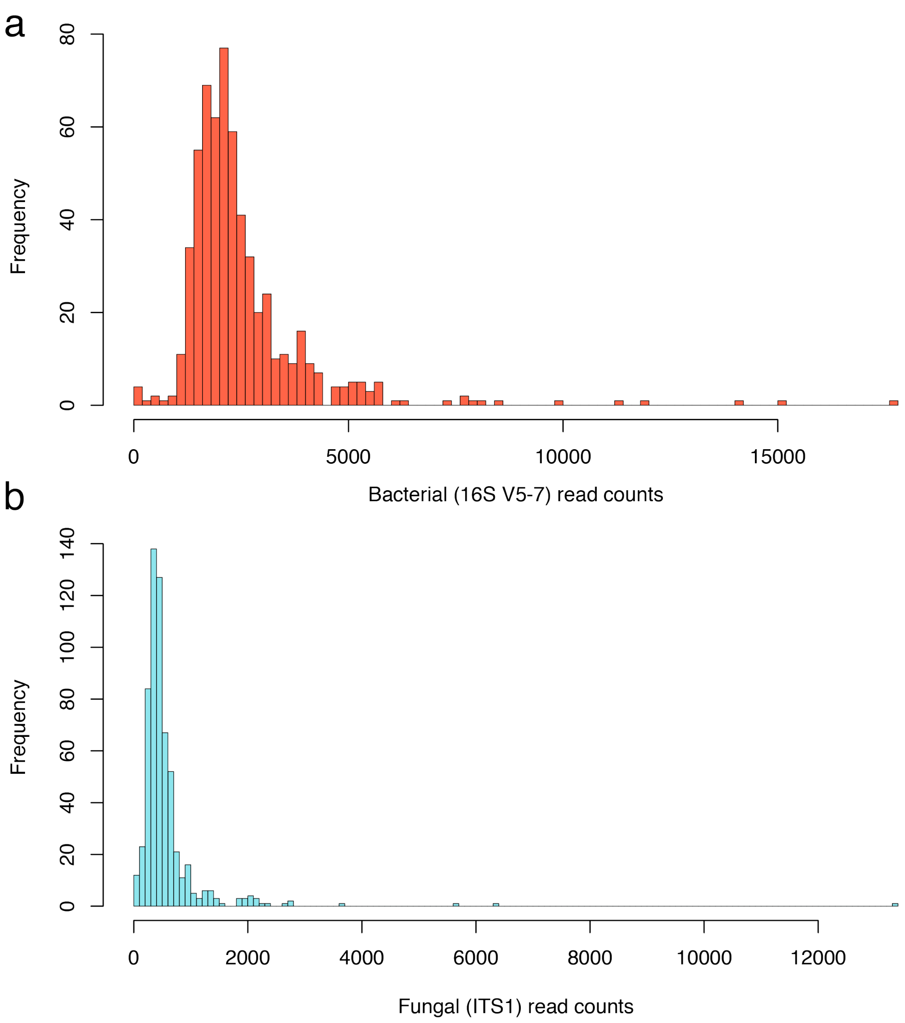

Supplementary Fig. S1. **Read counts per sample**

The number of reads per sample for root bacterial (a) and fungal (b) amplicon libraries.

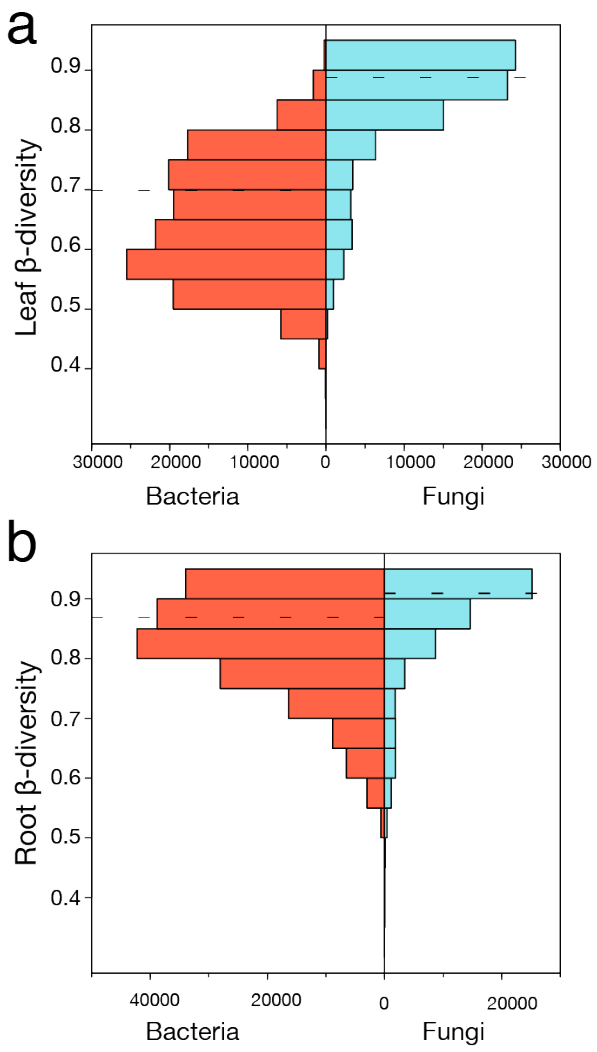

Supplementary Fig. S2. **β-diversity in the microbiome**

β-diversity in both the (a) leaf and (b) root microbiome of *A. thaliana*. On average, β-diversity is higher in the root than leaf microbiome for both kingdoms.

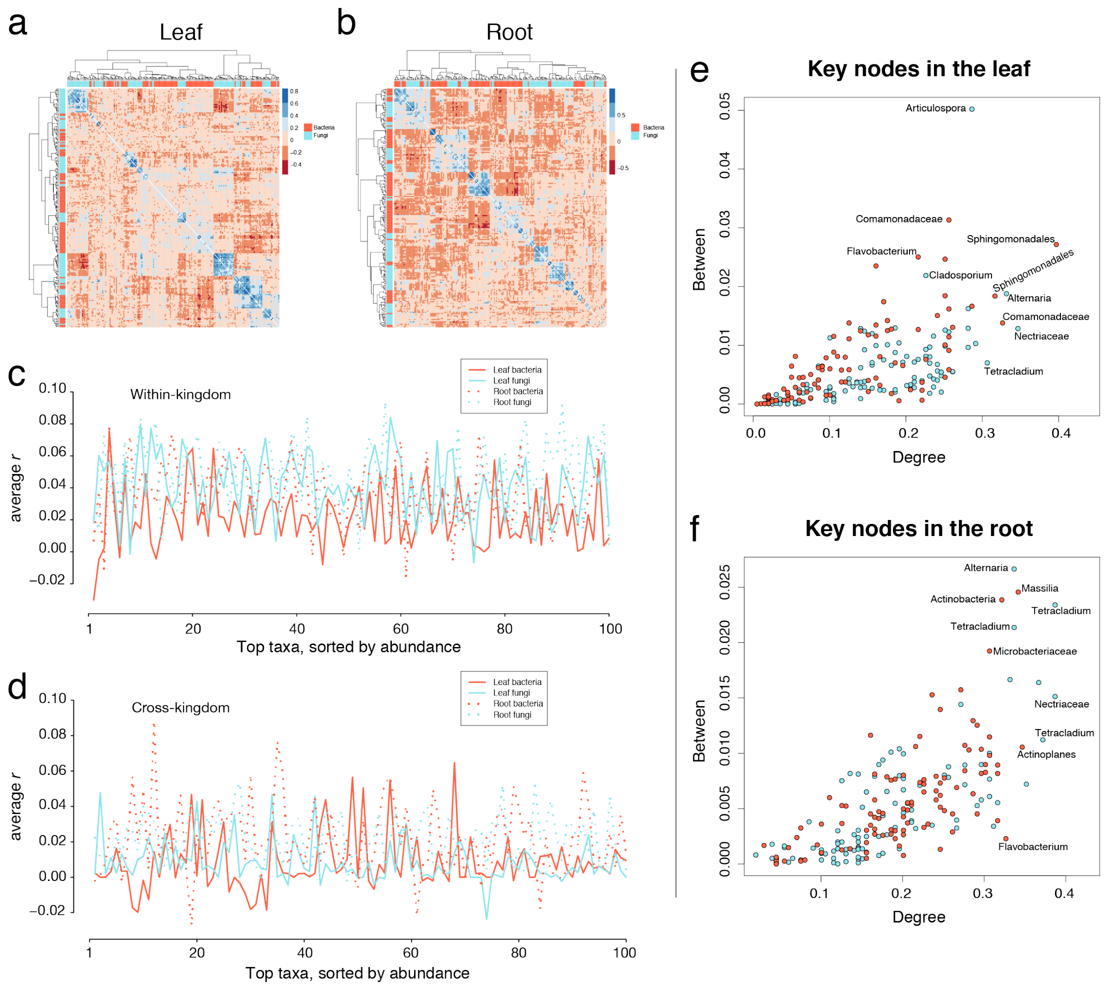

Supplementary Fig. S3. Key taxa in the microbiome

Correlations within the (a) leaf and (b) root microbiome of *Arabidopsis thaliana*. For ease of presentation and visualization, we show the top 100 taxa in each microbiome. As can be seen by the diagonal elements, the strongest correlations tend to occur among members of the same kingdom. Nevertheless, significant cross-kingdom correlations suggest that taxa within the leaf and root microbiome may interact or be shaped by similar environmental and/or host-related processes. (c) The average intra- and (d) inter-kingdom correlations for the top taxa in the plant microbiome show a slight positive skew. These Pearson correlations were calculated between each focal taxon (here, sorted based on their decreasing abundance) and their top (c) intra- or (d) inter-kingdom counterparts (that is, the comparisons are restricted to the top 100 taxa within each kingdom). (e) Network analysis helps to identify the key phylotypes in the leaf microbiome, which are shown here based on the centrality metrics *degree* and *betweenness*. (f) Influential nodes are shown for the root microbiome.

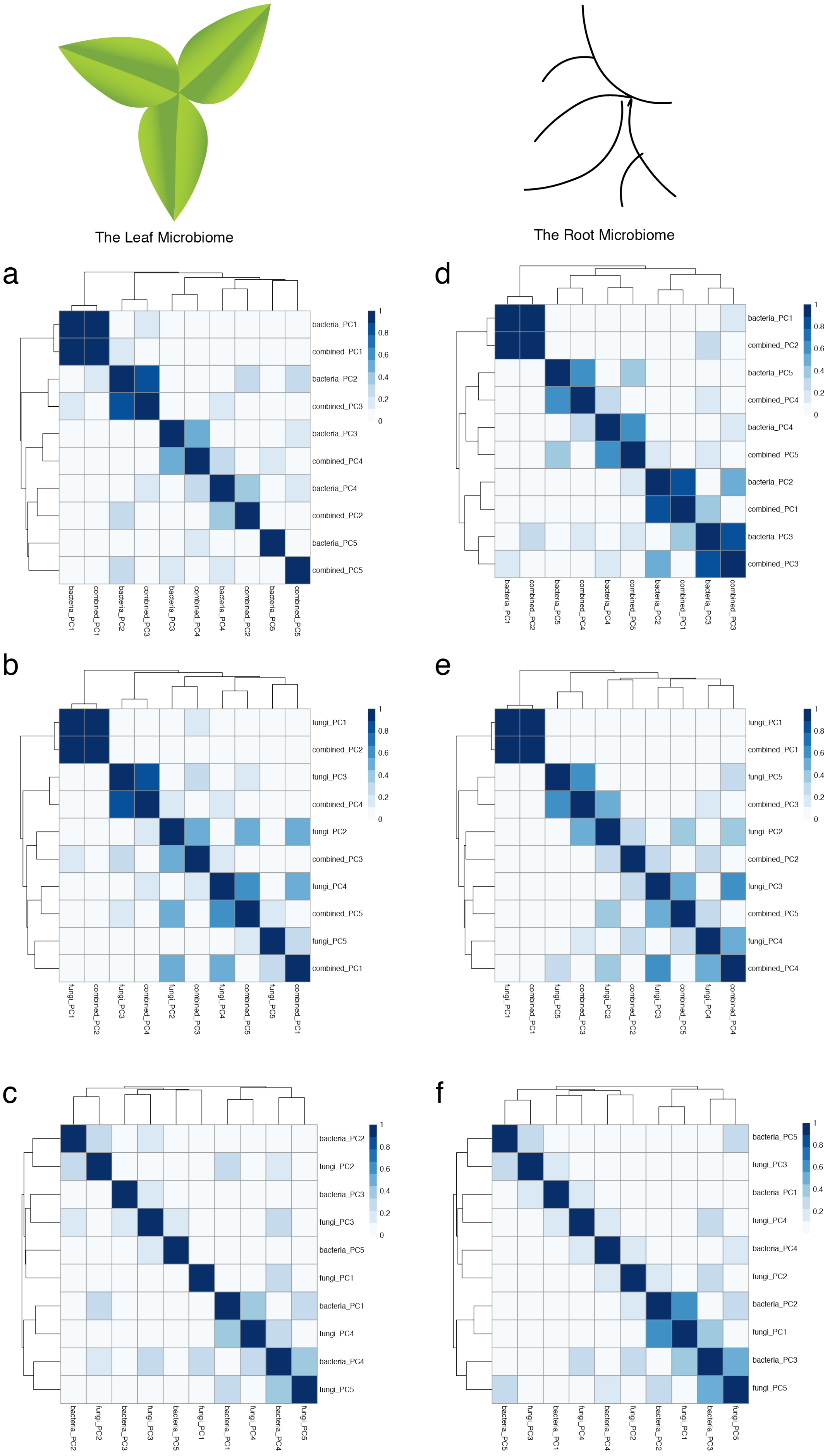
The Leaf Microbiome
The Root Microbiome
a
bacteria_PC1
combined_PC1
bacteria_PC2
combined_PC3
bacteria_PC3
combined_PC4
bacteria_PC4
combined_PC2
bacteria_PC5
combined_PC5
d
bacteria_PC1
combined_PC2
bacteria_PC5
combined_PC4
bacteria_PC4
combined_PC5
bacteria_PC2
combined_PC1
bacteria_PC3
combined_PC3
b
fungi_PC1
combined_PC2
fungi_PC3
combined_PC4
fungi_PC2
combined_PC3
fungi_PC4
combined_PC5
fungi_PC5
combined_PC1
e
fungi_PC1
combined_PC1
fungi_PC5
combined_PC3
fungi_PC2
combined_PC2
fungi_PC3
combined_PC5
fungi_PC4
combined_PC4
c
bacteria_PC2
fungi_PC2
bacteria_PC3
fungi_PC3
bacteria_PC5
fungi_PC1
bacteria_PC1
fungi_PC4
bacteria_PC4
fungi_PC5
f
bacteria_PC5
fungi_PC3
bacteria_PC1
fungi_PC4
bacteria_PC4
fungi_PC2
bacteria_PC2
fungi_PC1
bacteria_PC3
fungi_PC5
1
0.8
0.6
0.4
0.2
0

Supplementary Fig. S4. Correlations among the PCs from PCA of the microbiome

Correlation among the top 5 PCs from PCA of the leaf (a-c) and root (d-f) microbiome. PC1 and PC2 from PCA of the combined (bacteria and fungi) leaf-microbiome are correlated with PC1 from PCA of the bacterial community (a) and PC2 from PCA of the fungal community (b), respectively. (c) The correlation among the top 5 PCs from separate PCAs of the leaf bacterial and fungal community are shown. PC1 and PC2 from PCA of the combined root-microbiome are strongly correlated with PC1 (e) and PC2 (d) of the fungal and bacterial community. (f) Shown are the correlations among the top 5 PCs from PCA of the root bacterial and fungal community.

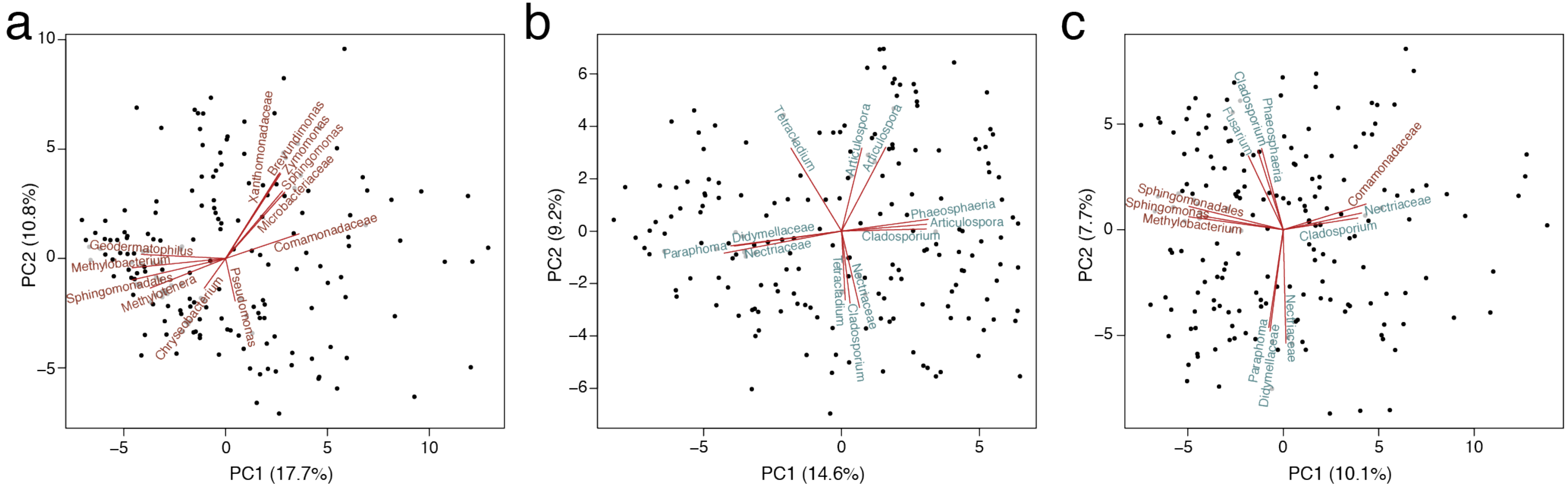

Supplementary Fig. S5. The results from PCA of the leaf microbiome

(a) A plot of principal component 1 (PC1) and PC2 from PCA of the top 100 bacteria in the leaf microbiome is shown. (b) A plot of PC1 and PC2 from PCA of the top 100 fungi in the leaf microbiome is shown. (c) A plot of PC1 and PC2 from PCA of the combined leaf microbial community, consisting of the top 100 bacteria and fungi. In each panel, the labels list the top 3 taxa whose abundances separate the samples along the axes (plotted according to their PC loadings).

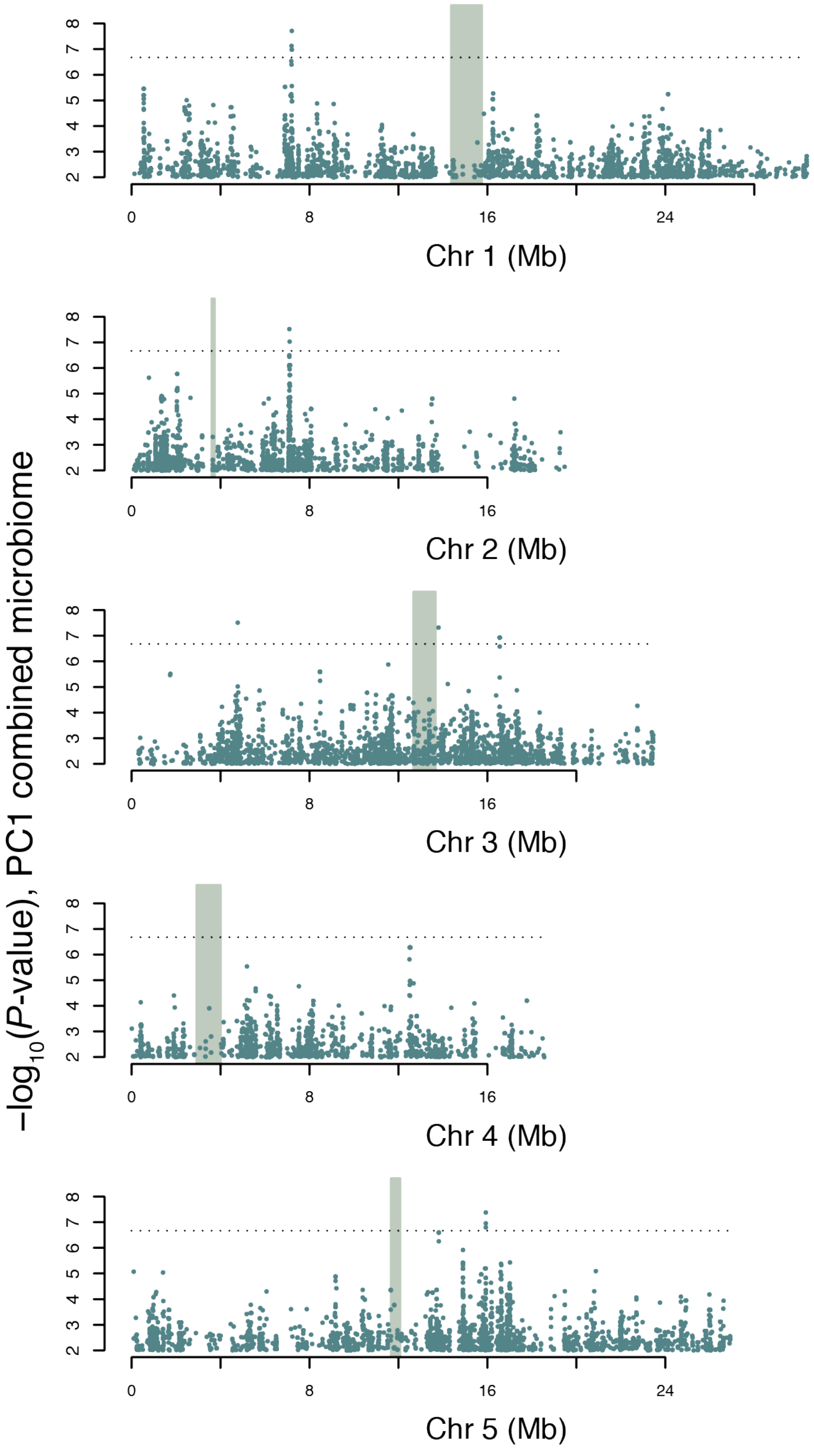

Supplementary Fig. S6. GWAS of PC1 from PCA of the combined microbiome

The results from GWAS of PC1 from PCA of the combined bacterial and fungal community are shown. The candidate genes are listed in the main text. To assess genome-wide significance, a permutation approach was used that takes into account population structure. The centromeres along each of *A. thaliana's* 5 chromosomes are shaded.

Supplementary Tables

Supplementary Table S1. The list of accessions used and their collection locations

Id	Name	Latitude	Longitude	Country
8251	Ag-0	45	1.3	France
8252	Alc-0	40.31	-3.22	Spain
8230	Algutsrum	56.68	16.5	Sweden
8253	An-1	51.2167	4.4	Belgium
8254	Ang-0	50.3	5.3	Belgium
8256	Ba1-2	56.4	12.9	Sweden
8257	Ba3-3	56.4	12.9	Sweden
8258	Ba4-1	56.4	12.9	Sweden
8259	Ba5-1	56.4	12.9	Sweden
8261	Bg-2	47.6479	-122.305	United States of America
8262	Bil-5	63.324	18.484	Sweden
8263	Bil-7	63.324	18.484	Sweden
8264	Bla-1	41.6833	2.8	Spain
8265	Blh-1	48	19	Czech Republic
8266	Boo2-1	55.86	13.51	Sweden
5837	Bor-1	49.4013	16.2326	Czech Republic
8268	Bor-4	49.4013	16.2326	Czech Republic
8269	Br-0	49.2	16.6166	Czech Republic
8231	Bro1-6	56.3	16	Sweden
8270	Bs-1	47.5	7.5	Switzerland
8271	Bu-0	50.5	9.5	Germany
8421	Buckhorn Pass	41.3599	-122.755	United States of America
8272	Bur-0	54.1	-6.2	Ireland
8274	Can-0	29.2144	-13.4811	Spain
8275	Cen-0	49	0.5	France
8276	CIBC-17	51.4083	-0.6383	United Kingdom
8277	CIBC-5	51.4083	-0.6383	United Kingdom
8278	Co	40.2077	-8.42639	Portugal
6909	Col-0			Unknown
8280	Ct-1	37.3	15	Italy
8233	Dem-4	41.1876	-87.1923	United States of America
8283	Dra3-1	55.76	14.12	Sweden
8284	DraII-1	49.4112	16.2815	Czech Republic
8285	DraIII-1	49.4112	16.2815	Czech Republic
6008	Duk	49.1	16.2	Czech Republic
6009	Eden-1	62.877	18.177	Sweden
8287	Eden-2	62.877	18.177	Sweden
8288	Edi-0	55.9494	-3.16028	United Kingdom

Id	Name	Latitude	Longitude	Country
6016	Eds-1	62.9	18.4	Sweden
8289	Ei-2	50.3	6.3	Germany
8290	En-1	50	8.5	Germany
8291	Est-1	58.3	25.3	Russia
8292	Fab-2	63.0165	18.3174	Sweden
8293	Fab-4	63.0165	18.3174	Sweden
8294	Fei-0	40.5	-8.32	Portugal
8422	Fja1-1	56.06	14.29	Sweden
6024	Fly2-2	55.7509	13.3712	Sweden
8295	Ga-0	50.3	8	Germany
8296	Gd-1	53.5	10.5	Germany
8297	Ge-0	46.5	6.08	Switzerland
8298	Got-22	51.5338	9.9355	Germany
8299	Got-7	51.5338	9.9355	Germany
8300	Gr-1	47	15.5	Austria
8301	Gu-0	50.3	8	Germany
8234	Gul1-2	56.4606	15.8127	Sweden
8303	H55	49	15	Czech Republic
8304	Hi-0	52	5	Netherlands
8235	Hod	48.8	17.1	Czech Republic
8423	Hov2-1	56.1	13.74	Sweden
8306	Hov4-1	56.1	13.74	Sweden
6039	Hovdala-2	56.1	13.74	Sweden
8308	HR-10	51.4083	-0.6383	United Kingdom
8309	HR-5	51.4083	-0.6383	United Kingdom
8310	Hs-0	52.24	9.44	Germany
8236	HSm	49.33	15.76	Czech Republic
8311	In-0	47.5	11.5	Austria
8312	Is-0	50.5	7.5	Germany
8313	Jm-0	49	15	Czech Republic
8314	Ka-0	47	14	Austria
8424	Kas-2	35	77	India
8237	Kavlinge-1	55.8	13.1	Sweden
8420	Kelsterbach-4	50.0667	8.5333	Germany
8238	Kent	51.15	0.4	United Kingdom
8316	Kin-0	44.46	-85.37	United States of America
6040	Kni-1	55.66	13.4	Sweden
8317	Kno-10	41.2816	-86.621	United States of America
8318	Kno-18	41.2816	-86.621	United States of America
8239	Koln	51	7	Germany
8319	Kondara	38.48	68.49	Tajikistan
8240	Kulturen-1	55.705	13.196	Sweden

Id	Name	Latitude	Longitude	Country
8320	Kz-1	49.5	73.1	Kazakhstan
8321	Kz-13	49.5	73.1	Kazakhstan
8322	Kz-9	49.5	73.1	Kazakhstan
8323	Lc-0	57	-4	United Kingdom
8324	Ler-1	47.984	10.8719	Germany
8241	Liarum	55.9473	13.821	Sweden
8242	Lillo-1	56.1494	15.7884	Sweden
8325	Lip-0	50	19.3	Poland
8326	Lis-1	56.0328	14.775	Sweden
8222	Lis-2	56.0328	14.775	Sweden
8430	Lisse	52.25	4.5667	Netherlands
8328	LL-0	41.59	2.49	Spain
8329	Lm-2	48	0.5	France
6042	Lom1-1	56.09	13.9	Sweden
6043	Lov-1	62.801	18.079	Sweden
6046	Lov-5	62.801	18.079	Sweden
8332	Lp2-2	49.38	16.81	Czech Republic
8333	Lp2-6	49.38	16.81	Czech Republic
8334	Lu-1	55.71	13.2	Sweden
8335	Lund	55.71	13.2	Sweden
8336	Lz-0	46	3.3	France
8337	Mir-0	44	12.37	Italy
8338	Mr-0	44.15	9.65	Italy
8339	Mrk-0	49	9.3	Germany
8340	Ms-0	55.7522	37.6322	Russia
8341	Mt-0	32.34	22.46	Libya
8342	Mz-0	50.3	8.3	Germany
8429	N13	61.36	34.15	Russia
8343	Na-1	47.5	1.5	France
8246	NC-6	35	-79.18	United States of America
8344	Nd-1	50	10	Switzerland
8345	NFA-10	51.4083	-0.6383	United Kingdom
8346	NFA-8	51.4083	-0.6383	United Kingdom
8347	Nok-3	52.24	4.45	Netherlands
8348	Nw-0	50.5	8.5	Germany
6064	Nyl-2	62.9513	18.2763	Sweden
8349	Omo2-1	56.1481	15.8199	Sweden
8350	Omo2-3	56.1481	15.8155	Sweden
6074	Or-1	56.4573	16.1408	Sweden
8351	Ost-0	60.25	18.37	Sweden
8352	Oy-0	60.23	6.13	Norway
8353	Pa-1	38.07	13.22	Italy

Id	Name	Latitude	Longitude	Country
8354	Per-1	58	56.3167	Russia
8355	Petergof	59	29	Russia
8243	PHW-2	43.7703	11.2547	Italy
8244	PHW-34	48.6103	2.3086	France
8356	Pi-0	47.04	10.51	Austria
8358	Pna-10	42.0945	-86.3253	United States of America
8359	Pna-17	42.0945	-86.3253	United States of America
8360	Pro-0	43.25	-6	Spain
8361	Pu2-23	49.42	16.36	Czech Republic
8362	Pu2-7	49.42	16.36	Czech Republic
8363	Pu2-8	49.42	16.36	Czech Republic
8364	Ra-0	46	3.3	France
8365	Rak-2	49	16	Czech Republic
8366	Rd-0	50.5	8.5	Germany
8367	Ren-1	48.5	-1.41	France
8368	Ren-11	48.5	-1.41	France
8369	Rev-1	55.6942	13.4504	Sweden
8370	Rmx-A02	42.036	-86.511	United States of America
8371	Rmx-A180	42.036	-86.511	United States of America
8372	RRS-10	41.5609	-86.4251	United States of America
8373	RRS-7	41.5609	-86.4251	United States of America
8374	Rsch-4	56.3	34	Russia
8375	Rubezhnoe-1	49	38.28	Ukraine
8247	San-2	56.07	13.74	Sweden
8376	Sanna-2	62.69	18	Sweden
8377	Santa Clara	37.21	-121.16	United States of America
8378	Sap-0	49.49	14.24	Czech Republic
8412	Sav-0	49.1833	15.8833	Czech Republic
8379	Se-0	38.3333	-3.53333	Spain
8245	Seattle-0	47	-122.2	United States of America
8380	Sf-1	41.7833	3.03333	Spain
8248	Shahdara	38.35	68.48	Tajikistan
8381	Sorbo	38.35	68.48	Tajikistan
8382	Spr1-2	58.4168	14.1612	Sweden
8383	Spr1-6	58.4168	14.1612	Sweden
8384	Sq-1	51.4083	-0.6383	United Kingdom
8385	Sq-8	51.4083	-0.6383	United Kingdom
8386	Sr:5	58.9	11.2	Sweden
8387	St-0	59	18	Sweden
6088	Stu1-1	56.4666	16.1284	Sweden
8388	Stw-0	52	36	Russia
8389	Ta-0	49.5	14.5	Czech Republic

Id	Name	Latitude	Longitude	Country
8390	Tamm-2	60	23.5	Finland
8391	Tamm-27	60	23.5	Finland
6243	Tottarp-2	55.95	13.85	Sweden
8392	Ts-1	41.7194	2.93056	Spain
8393	Ts-5	41.7194	2.93056	Spain
8394	Tsu-1	34.43	136.31	Japan
8395	Tu-0	45	7.5	Italy
8426	Ull1-1	56.06	13.97	Sweden
8396	Ull2-3	56.0648	13.9707	Sweden
8397	Ull2-5	56.0648	13.9707	Sweden
8398	Uod-1	48.3	14.45	Austria
8428	Uod-2	48.3	14.45	Austria
8399	Uod-7	48.3	14.45	Austria
8400	Van-0	49.3	-123	Canada
8401	Var2-1	55.58	14.334	Sweden
8402	Var2-6	55.58	14.334	Sweden
9058	Vastervik	57.75	16.6333	Sweden
8249	Vimmerby	57.7	15.8	Sweden
9057	Vinslov	56.1	13.9167	Sweden
8403	Wa-1	52.3	21	Poland
8404	Wei-0	47.25	8.26	Switzerland
8405	Ws-0	52.3	30	Russia
8406	Ws-2	52.3	30	Russia
8407	Wt-5	52.3	9.3	Germany
8408	Yo-0	37.45	-119.35	United States of America
8409	Zdr-1	49.3853	16.2544	Czech Republic
8410	Zdr-6	49.3853	16.2544	Czech Republic

Table S2, The biological processes that underlie variation in richness in the root microbiome

The biological (gene ontology, GO) processes enriched in the 1% tail from GWAS of species richness in the bacterial and fungal community are shown. Storey's procedure was used to correct for multiple testing.

Kingdom	Biological process	Enrichment	FDR $q \leq 10\%$
fungi	translesion synthesis	77.4	0.0002
fungi	zeta DNA polymerase complex	77.4	0.0002
fungi	alpha-glucosidase activity	44.2	0.0011
fungi	epidermal cell differentiation	16.6	0.0025
fungi	structural constituent of ribosome	2.2	0.0443
fungi	cytosolic large ribosomal subunit	2.9	0.0536
fungi	DNA repair	3.1	0.0868
fungi	response to UV	5.4	0.0868
fungi	microtubule motor activity	3.2	0.0868
fungi	ribosome	2.2	0.0972
fungi	translation	2.0	0.0972
fungi	root epidermal cell differentiation	6.4	0.0972
fungi	programmed cell death	6.3	0.0972
fungi	cytosolic ribosome	2.2	0.0975
bacteria	tRNA dimethylallyltransferase activity	59.5	0.00004
bacteria	cell wall modification involved in multidimensional cell growth	44.2	0.0020
bacteria	galactose catabolic process	28.1	0.0046
bacteria	homoserine dehydrogenase activity	28.1	0.0046
bacteria	cellulase activity	22.1	0.0047
bacteria	phosphoglucomutase activity	22.1	0.0047
bacteria	cytosolic part	22.1	0.0047
bacteria	glucose 1-phosphate metabolic process	22.1	0.0047
bacteria	cytokinin biosynthetic process	12.2	0.0047

bacteria	tRNA processing	11.9	0.0047
bacteria	aspartate family amino acid biosynthetic process	18.2	0.0066
bacteria	detection of gravity	17.2	0.0072
bacteria	gametophyte development	11.0	0.0249
bacteria	protein import into nucleus	8.8	0.0440
bacteria	cotyledon vascular tissue pattern formation	8.3	0.0480
bacteria	starch biosynthetic process	7.5	0.0600
bacteria	SCF ubiquitin ligase complex	4.7	0.0793
bacteria	cellular response to phosphate starvation	2.9	0.0793
bacteria	ubiquitin-protein ligase activity	2.1	0.0793
bacteria	pectinesterase inhibitor activity	3.6	0.0889
bacteria	protein ubiquitination	3.1	0.0948
